# Cognition and emotional distress in middle-aged and older adults with spina bifida myelomeningocele

**DOI:** 10.1371/journal.pone.0298891

**Published:** 2024-02-29

**Authors:** Elisabeth Fagereng, Ingeborg Beate Lidal, Kerstin Lundberg Larsen, Marianne Løvstad, Tiina Rekand, Solveig Lægreid Hauger

**Affiliations:** 1 TRS National Resource Centre for Rare Disorders, Sunnaas Rehabilitation Hospital, Bjørnemyr, Norway; 2 Department of Research, Sunnaas Rehabilitation Hospital, Bjørnemyr, Norway; 3 Department of Psychology, Faculty of Social Sciences, University of Oslo, Oslo, Norway; 4 Department of Neurology, Haukeland University Hospital, Bergen, Norway; Ohio State University, UNITED STATES

## Abstract

**Purpose:**

To investigate cognitive functioning and emotional distress in adults aged 55 to 68 years old with spina bifida myelomeningocele (SBM), both with and without hydrocephalus. A secondary aim was to explore the associations between psychosocial factors in relation to emotional distress.

**Materials and methods:**

Cross-sectional study of eleven females and eight males with SBM, five with and twelve without hydrocephalus. Cognitive functioning was investigated with neuropsychological tests and self-report measures. Furthermore, participants completed questionnaires regarding resilience, access to social support, coping, and emotional distress. Descriptive statistics were applied, and Spearman Rho correlation coefficients were used to explore the relationships between psychosocial factors and emotional distress.

**Results:**

Eleven exhibited normal cognitive functioning. An observed difference was seen between participants with and without hydrocephalus, where six and five persons reported clinical levels of depression and anxiety, respectively. Positive perceptions of self and future were associated with lower levels of depression and anxiety.

**Conclusion:**

This study adds important information about cognitive functioning and emotional distress in an understudied population. The results indicated normal cognitive functioning in adults aged 55 to 68 years with SBM without hydrocephalus. Prevalence of emotional distress was comparable with previous studies of younger adults with SBM. There is a need for longitudinal studies investigating cognition and psychological health to fully capture important aspects of the life course of SBM with and without hydrocephalus.

## Introduction

Spina bifida (SB) encompasses a heterogeneous group of congenital neural tube defects. SB myelomeningocele (SBM) is the most common and severe form due to neurological symptoms such as paralysis, neurogenic bladder and bowel, and cognitive deficits [1]. The presence of neuroanatomical abnormalities in SBM varies, but approximately 85% develop hydrocephalus and most have Chiari malformation type II [2]. In addition, about 50% display partial dysgenesis or hypoplasia of the corpus callosum [3]. These abnormalities to the brain are significant determinants of cognitive outcome in SBM [2]. Importantly, prior to the introduction of valved shunt as standard treatment for hydrocephalus during the 1960s, most persons with SBM and hydrocephalus did not survive into adulthood [1]. It has been shown very high mortality rates in SBM at younger age before 1975, and an increased survival in western countries more recently [4]. However, survival and consequences of SBM into middle- (i.e., 40–60 years) and older age (i.e., 60 years and older) have not yet been systematically studied. Hence, little is known with regard to cognitive, physical and psychological health in persons whom have lived with SBM for more than five decades.

In addition, the prevalence of people with SBM with older age is unknown. However, with medical advancement and increased birth survival rate, one would expect more persons with SBM approaching adulthood and becoming old, thus knowledge of this population is needed. A previous study from our research group calculated that in Norway, with about 5.4 million inhabitants, fewer than 100 persons with SBM aged 50 years or above, were alive in 2016 [5]. In that study, which this paper is based on, Lidal et al. [5] investigated physical function, participation and health issues in 30 community-dwelling persons with SBM aged 50 years or older. The majority of them reported perceived deterioration of mobility along with other secondary health issues such as obesity, hypertension and pain [5]. Another longitudinal study of 37 adults with SBM born between 1963 and 1971 in the United Kingdom also showed gradual decline in mobility with increasing age [6]. While somatic and physical functioning have been explored somewhat in the SBM population ageing into middle-age [5–7], few studies have addressed cognitive and psychological functioning in persons aged 55 years and older.

### Cognitive functioning in SBM

Varying degrees of cognitive impairment frequently accompany SBM from infancy and throughout the lifetime [8]. Typically, deficits are commonly seen in the domains of processing speed, attention, visuospatial function, prospective memory and executive function [9–12]. A relative impairment in performance IQ compared to verbal IQ is also often observed [3]. The conceptualization of a typical cognitive profile is however mainly based on studies of children, adolescents and young adults. For example, a recently published systematic review by Sachdeva et al. [13], reported deficits in working memory, attention and prospective memory, but none of the 24 studies in the review included samples with a median age above 35 years.

Nearly all studies on cognitive functioning in adults with SBM include participants with additional brain abnormalities [13]. While the majority of persons with SBM born after the 1960`s exhibit brain abnormalities such as hydrocephalus, Chiari malformation type II, and corpus callosum dysgenesis, a larger proportion of those who were born prior to the introduction of valved shunt treatment and survived into late adulthood, do not have these complications. There is a need for more knowledge regarding cognitive functioning in middle-aged and older adults with SBM, which will also result in a population where the majority did not have severe brain abnormalities.

### Emotional distress, resilience, and social support in SBM

Although psychological functioning in SBM is mostly studied in children and adolescents [14], studies on adults indicate an elevated risk for depression and anxiety compared to the general population [14, 15], and prevalence estimates vary between 18–48% [15–19] for depression and 17–47% for anxiety [15, 16, 18, 19]. However, none of these studies included samples with a mean age above 40 years and are heterogeneous in terms of SB sample and measurement tools, leading to high variability in prevalence rates. In a study investigating depression and anxiety in 30 middle-aged and older persons with SBM in Norway, Lidal et al. [20] found that 30% and 20% scored above the thresholds for clinically relevant symptoms of anxiety and depression measured with Hospital Anxiety and Depression Scale (HADS). In contrast, a large population-based study in Norway using HADS reported that 8.6% and 14.9% of adults aged 50 to 79 years old met the criteria for clinically relevant depression and anxiety, respectively [21].

Existing studies on emotional functioning in SBM have not focused on potentially protective factors that buffer against emotional problems. The mental health guidelines for SB from 2020 [22] highlights encouragement of activities, social engagement and promotion of goals and pursuit in prevention of mental health issues. While social support is shown to buffer against emotional distress in children with SB [23, 24], social support has not been examined in the adult population. Despite an increasing interest in resilience factors in disability research, research targeting resilience in SBM is limited. Two prior studies found that resilience was associated with higher age, better physical functioning and mental health [19, 25]. Also, while we know that the use of active coping strategies as opposed to strategies related to emotional and behavioural avoidance, are associated with better psychosocial adaption in chronic disease [26], coping has largely not been addressed in adults with SBM. One study by Stubberud et al. [27] demonstrated that seven weeks of cognitive training, using Goal Management Training, resulted in a significant increase in task focused coping and a decrease in avoidant coping in 24 adults with SBM aged between 19 and 45 years.

Overall, SBM is a heterogeneous condition, and we know little about long-term cognitive and emotional outcomes in the limited number of middle aged and older SBM survivors. The primary aim of the current study was therefore to describe cognitive function and emotional distress in middle-aged and older adults with SBM. A secondary aim was to explore the associations between psychosocial factors, i.e. resilience, access to social support and coping in relation to emotional distress. 

## Materials and methods

### Participants and procedure

The target population in the current cross-sectional study was the same 30 persons (18 females and 12 males) from all parts of Norway with SB who participated in the Lidal et al. study in 2017 [5]. This previous study recruited persons with SB born prior to 1967 who were registered at TRS National Resource centre for Rare Disorders at Sunnaas Rehabilitation Hospital, Norway. In addition, recruitment was conducted through announcement on the website of the Norwegian Association for Spina Bifida and Hydrocephalus (n = 62). The exclusion criteria were psychiatric conditions that interfered with the ability to provide informed consent or co-morbid severe somatic illness that would interfere with participation. The cohort represented about 30% of persons with SBM aged 50+ alive in Norway in 2017 [5]. At the time of inclusion in the current study, four persons from the original cohort were deceased, leaving a total of 26 whom received a letter of invitation to participate primo 2021. A short telephone interview was undertaken by one researcher to ensure that responders understood the study information. The participants were invited to face-to-face outpatient interviews and examinations at Sunnaas Rehabilitation Hospital between May and November 2021. Four participants were precluded to travel a long distance to the hospital due to health issues and were offered a home visit. The data collection took approximately 4–6 hours per participant and was conducted by a physician (IBL), physiotherapist (KLL) and psychologist (EF*). Only these had access to information that could identify individual participants during or after data collection.

### Ethical considerations

The study was approved by the Regional Ethic Committee for Medical Research Ethics (REK-number 211538), South-Eastern Norway. All participants provided written informed consent. The research was completed in accordance with the Helsinki Declaration. Participants reporting suicidal ideations were given a consultation to assess the need for further follow-up.

### Measurements

#### Demographics and medical variables

Participant characteristics included sex, age, employment status, and years of education. Medical variables included self-reported SB level (sacral, lumbar, and thoracic), presence of hydrocephalus, shunt status, and presence of Chiari II malformation. Ambulation status was assessed by a study physiotherapist and categorised as 1 = persons who walked independently (without aids), 2 = persons who walked with the use of aids, and 3 = persons with no functional walking ability.

#### Cognitive functioning

Cognitive functioning was assessed with commonly used test measures in clinical neuropsychology. An overview of the cognitive domains and specific test measures used are reported in Table 1. To characterize the level of cognitive functioning in the sample, the verbal test Similarities and the non-verbal tests Matrix Reasoning and Block Design from the Wechsler Adult Intelligence Scale Fourth Edition (WAIS-IV) [28] were included. In addition, visuo-motor coordination was assessed with The Grooved Pegboard from the Halstead-Reitan Neuropsychological Battery [29]. Psychomotor speed and mental flexibility were assessed with the Trail Making Test (TMT) 2, 3, and 4 from The Delis-Kaplan Executive Function System (D-KEFS) [30]. The Verbal Fluency Test from D-KEFS [30] was also included, which measures the ability to assess word generation and cognitive flexibility, and the Color Word Interference Test (CWIT) from D-KEFS [30] for assessing inhibition and mental flexibility. Attention span and working memory were assessed with the Digit Span from WAIS-IV [28], while sustained attention, vigilance, inattentiveness, and inhibition was assessed with the Continuous Performance Test, 3rd. edition [31].

**Table 1:** Overview of neuropsychological tests and cognitive domains.

Neuropsychological test	Cognitive domain
Grooved Pegboard (HRNB)	Visual-motor coordination
Trail Making Test (TMT; D-KEFS)	
• TMT 2	Psychomotor speed
• TMT 3	Psychomotor speed
• TMT 4	Psychomotor speed, mental flexibility
Verbal Fluency Test (D-KEFS)	
• Letter Fluency	Phonemic word generation
• Category Fluency	Categorical word generation
• Category Switching	Categorical word generation and mental flexibility
Color Word Interference Test (CWIT; D-KEFS)	
• Color Naming	Mental efficiency
• Word Reading	Mental efficiency
• Inhibition	Inhibition
• Inhibition Switching	Inhibition and mental flexibility
Similarities (WAIS-IV)	Abstract verbal reasoning
Block Design (WAIS-IV)	Visuospatial perception
Matrix Reasoning (WAIS-IV)	Non-verbal abstract problem solving
Digit Span (DS; WAIS-IV)	
• DS Forward	Auditory attention span
• DS Backward	Auditory working memory
• DS Sequencing	Auditory working memory
CPT-3	Sustained attention, vigilance, inattentiveness and inhibition

HRNB: Halstead-Reitan Neuropsychological Battery; D-KEFS: The Delis-Kaplan Executive Function System; WAIS-IV: Wechsler Adult Intelligence Scale– 4th. edition; CPT-3: Continuous Performance Test, 3rd. edition

In addition to performance-based measures, self-reported measures were used to investigate subjective cognitive functioning. Self-reported executive functioning was measured using the Behavior Rating Inventory of Executive Function–Adult version (BRIEF-A). The BRIEF-A is a standardized questionnaire that measures self-reported executive functions in everyday life, developed by Gioia and colleagues in 2000 [32]. Based on nine sub-scales, the BRIEF-A provides an overall Global Executive Composite (GEC) score and two Composite Index scores: the Behavioral Regulation Index (BRI) and the Metacognition Index (MI). Statements are answered on a three-point Likert Scale ranging from 1 (never) to 3 (often) reflecting degree of occurrence during the past 6 months. The BRIEF-A has demonstrated high reliability, with an internal consistency of Cronbach’s alpha value of .94 and .96 for BRI and MI respectively, and is considered an ecologically sensitive measure of executive functioning [32, 33].

In addition to the BRIEF-A, three items in the cognitive subscale from the Rivermead Post-Concussion Questionnaire (RPQ) [34] were used to measure self-reported memory, attention and mental speed. On the RPQ, each item is scored on a five-point Likert scale ranging from 0 (not experienced at all) to 4 (a severe problem). The instruction was adapted to SB by adjusting the timeframe to within the last week.

#### Emotional distress

Depressive symptoms were measured with the depression module of the Patient Health Questionnaire (PHQ-9), which covers the nine diagnostic criteria for depressive disorder in the Diagnostic and Statistical Manual of Mental Disorders (DSM-IV) [35]. Each item is scored on a four-point Likert scale ranging from 0–3. In line with recommendations, a score of 10 or above indicates clinically relevant depression [36]. The PHQ-9 is regarded as a valuable tool in diagnosing depression as well as a reliable and valid measure of depression severity, showing an internal consistency of 0.87, sensitivity of 88% and specificity of 85% [36, 37].

Anxiety symptoms were measured with the 7-item Generalized Anxiety Disorder (GAD-7) questionnaire [38]. The GAD-7 was designed to assess symptoms during the previous two weeks, and has been validated as an anxiety screening tool and severity measure in different populations with good internal consistency ranging from 0.83 to 0.93 [38, 39]. The GAD-7 consist of seven items which are answered on a four-point Likert scale. The scores range from 0 to 21. A score of 10 is proposed as threshold for clinically relevant anxiety [38].

#### Psychosocial factors

Coping was measured using the Brief COPE Inventory (Brief Cope) [40]. The Brief Cope includes 28 items to be answered on a four-point Likert scale ranging from 1–4, in which higher scores indicate increased utilization of the coping strategy in question. The 14 subscales in the original scale were grouped into four coping strategies based on the 4-factor structure proposed by Baumstarck et al. [41]: seeking social support, problem solving, avoidance, and positive thinking. The 4-factor structure has been shown to have satisfactory psychometric properties and internal consistency ranging from 0.71 to 0.82 [41].

Resilience was measured with the Resilience Scale for Adults (RSA) [42], which is a self-report scale designed to measure factors associated with resilience in three domains; dispositional, social and external resources. The RSA consists of 33 items and includes six subscales: perceptions of self, perception of future, social competence, family cohesion, social resources and structured style. The items are answered on a seven-point Likert scale where higher scores indicate higher degree of resilience. The RSA has shown good psychometric properties, with internal consistency estimates on the subscales varying from 0.67–0.90 [42].

Social support was assessed using the Duke-UNC Functional Social Support Questionnaire (FSSQ) [43]. The FSSQ incorporates eight items that are answered on a five-point Likert scale ranging from 1–5. Higher scores indicate greater perceived social support. The FSSQ is widely used in research on both somatic and psychiatric illnesses, and is reported to have good psychometric properties and Cronbach alpha level of 0.92 [43, 44].

### Statistical analyses

Descriptive statistics were applied to depict frequencies. Due to a small sample size, descriptive statistics are reported in both median (Mdn) with 1st and 3rd quartile (Q1, Q3), mean (M) with standard deviation (SD), and range. Spearman Rho correlation coefficients were used to explore the relationship between psychosocial factors and emotional distress. The strength of the correlation coefficients are interpreted according to Cohen’s guidelines, i.e. *r* = .10, *r* = .30, and *r* = .50 are considered small, medium, and large in magnitude, respectively [45]. Participants with and without hydrocephalus are reported separately.

For the neuropsychological tests, raw scores were transformed into age-equivalent T-scores (M = 50; SD = 10). Higher values indicate better performance relative to age-specific norms for all tests except from CPT-3, where lower values indicate better performance. Neuropsychological test performance was classified as “normal” if T-scores fell within 1½ SD from the mean [46]. With regard to the BRIEF-A, age-corrected T-scores (M = 50; SD = 10) are provided for each index and subscale, in which clinically elevated scores were defined as a T-score of 65 or greater, in line with the manual [32]. The BRIEF-A operates with three validity scales with recommended cut-off values (Negativity >6, Infrequency >3, and Inconsistency >8) [32]. The BRIEF-A data from one participant was excluded due to an elevated score on the Inconsistency scale.

Missing data on questionnaires were handled by imputing the missed item with the mean. Statistical analyses were carried out in November 2022 using SPSS 28.0 (IBM Inc., Armonk, NY, USA). Statistical significance was defined as an alpha-level of ≤.05.

## Results

### Demographic and clinical results

**Fig 1:**
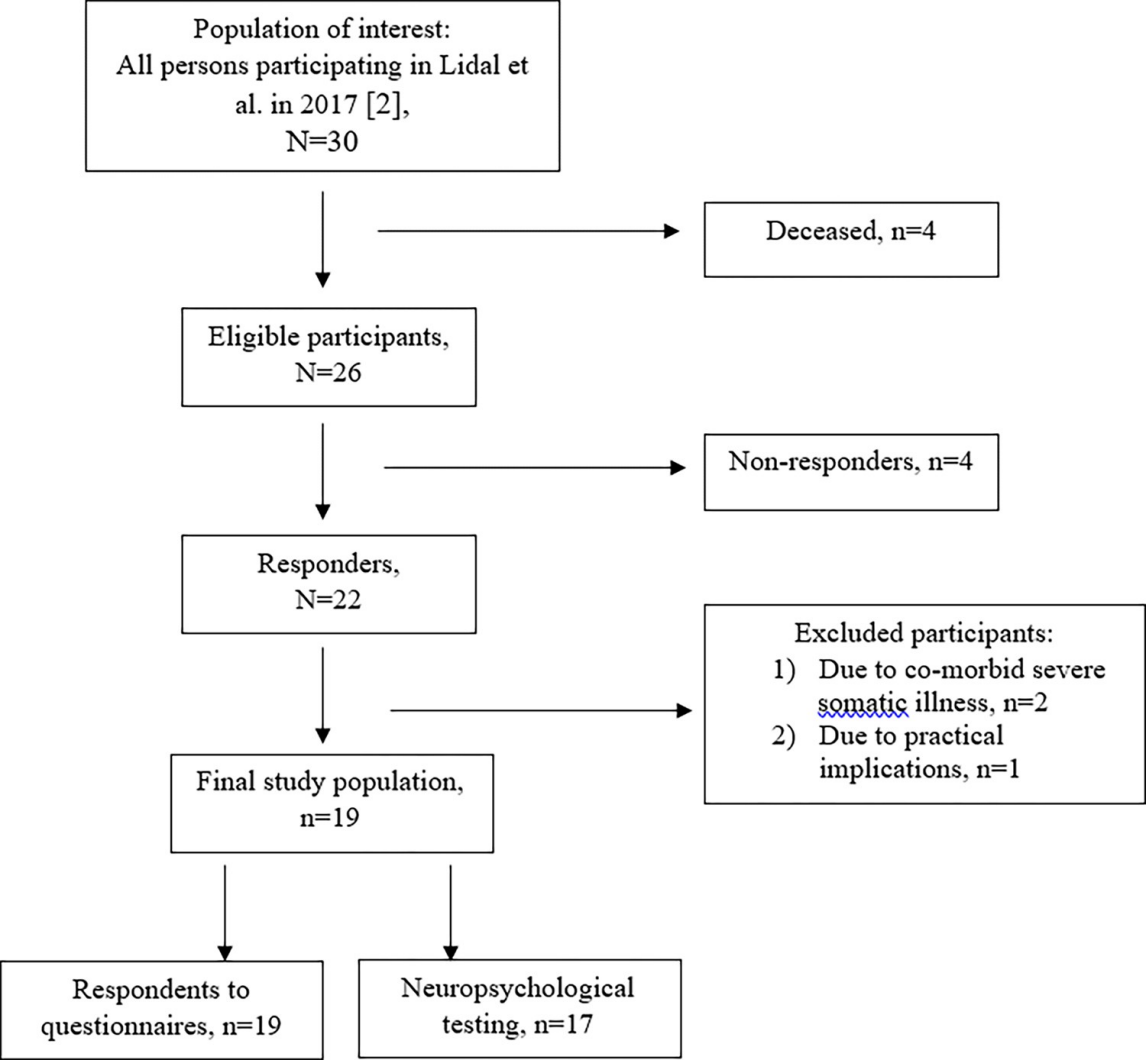
Flow chart.

Of the 26 eligible participants, four did not return the informed consent letter, and three were excluded due to severe comorbid health conditions or practical implications. See Fig 1 for flow chart. The current study sample thus consists of 19 adults with SBM aged 55–68 years from the original cohort of 30. All responded to questionnaires, except from one missing the RSA and two missing the RPQ. Seventeen participants completed neuropsychological testing, although not all 17 were able to complete the full test battery.

Demographic and clinical variables are presented in Table 2. The median age was 60 years (range = 55–68). A slight majority was female (n = 11), received full disability benefits (n = 11), and had finished upper secondary school (n = 11). Furthermore, most had lumbar SB level (n = 12) and about half of them used walking aids for ambulation (n = 10). Only five participants had hydrocephalus, all treated with a shunt.

**Table 2:** Demographics: Sex, age, education, occupational status. Clinical: Lesion level, hydrocephalus, ambulation status.

	Total sample (N = 19)		
	N/Mean (SD)	Median [Q1, Q3]	Range
Demographics			
• Sex, female/male	11/8		
• Age	60.4 (3.7)	60 [58, 64]	55–68
• Education, years	12.6 (2.9)	12 [12, 15]	9–19
• Primary	3		
• Upper secondary	11		
• Higher education	5		
• Occupational status			
• Full time	3		
• Part time	4		
• Full disability benefits^a^	11		
Clinical			
• Lesion level			
• Sacral	7		
• Lumbar	12		
• Hydrocephalus	5		
• Ambulation status			
• Independently walker	3		
• Walkers with aids	10		
• Non-walker	6		

^a^One person was retired

### Cognitive functioning

The neuropsychological test results are summarized in Table 3. Mean test scores in the total sample fell within the normal range for each test, i.e., the group mean was within 1½ SD from the normative mean. The group without hydrocephalus obtained neuropsychological mean test scores within the normal range. In the group of five participants with hydrocephalus, however, mean test scores varied ranging from below to within normal range. At an individual level, three out of twelve participants in the group without hydrocephalus had two or more test scores below normal range (t-score <35), while four out of five participants with hydrocephalus displayed neuropsychological test performance below normal range on two or more tests.

**Table 3:** Results from neuropsychological tests.

	Total sample (N = 17)	Without hydrocephalus (N = 12)	With hydrocephalus (N = 5)
	Mean (SD)	Mean (SD)	Mean (SD)
Grooved Pegboard (HRNB)			
• Grooved Pegboard right, *n = 16*	40.88 (14.62)	46.67 (10.77)	23.50 (10.34)
• Grooved Pegboard left, *n = 16*	42.44 (11.55)	46.33 (9.20)	30.7 (10.69)
Trail Making Test (TMT; D-KEFS)			
• TMT 2, n = 15	48.93 (13.56)	54.00 (10.29)	35.00 (12.36)
• TMT 3, n = 15	51.00 (10.71)	54.45 (6.67)	41.50 (14.93)
• TMT 4, n = 14	49.50 (7.64)	49.09 (8.63)	51.00 (1.73)
Verbal Fluency (VF; D-KEFS)			
• VF Letter Fluency	54.76 (10.35)	58.33 (9.54)	46.20 (7.00)
• VF Category Fluency	56.29 (12.91)	62.25 (8.75)	42.00 (9.72)
• VF Category Switching Correct …responses	50.18 (10.60)	54.42 (8.98)	40.00 (6.63)
• VF Category Switching accuracy	51.29 (12.14)	56.33 (8.04)	39.20 (12.38)
Color Word Interference Test (CWIT; D-KEFS)			
• CWIT 1, n = 16	46.88 (13.00)	51.67 (7.87)	32.50 (15.84)
• CWIT 2, *n = 16*	48.75 (10.98)	53.67 (5.50)	34.00 (10.23)
• CWIT 3, *n = 16*	50.75 (11.65)	56.08 (3.23)	34.75 (13.62)
• CWIT 4, *n = 15*	50.47 (7.54)	52.75 (6.25)	41.33 (5.13)
Similarities (WAIS-IV)	47.18 (8.93)	49.17 (8.01)	42.40 (10.09)
Matrix Reasoning (WAIS-IV)	49.47 (10.88)	51.42 (6.02)	44.80 (18.31)
Digit Span (DS; WAIS-IV)			
• DS Forward	44.41 (9.69)	46.83 (9.67)	38.60 (7.67)
• DS Backwards	46.47 (8.51)	45.25 (9.87)	49.40 (2.51)
• DS Sequencing	45.35 (8.12)	46.75 (8.51)	42.00 (6.67)
Continuous Performance Test, 3^rd^. edition (CPT-3)^a^			
• CPT-3 Omissions, *n = 14*	52.79 (14.15)	50.45 (13.23)	61.33 (16.92)
• CPT-3 Commissions, *n = 14*	47.64 (8.04)	45.73 (8.01)	54.67 (2.08)
• CPT-3 HRT, *n = 14*	57.71 (5.92)	56.82 (5.83)	61.00 (6.08)
• CPT-3 HRT Block Change, *n = 14*	53.21 (7.59)	54.27 (8.05)	49.33 (4.62)

Neuropsychological Battery; D-KEFS: The Delis-Kaplan Executive Function System; WAIS-IV: Wechsler Adult Intelligence Scale– 4th. ed; HRT: Hit Reaction Time.

^a^Lower scores on CPT-3 indicate better performance.

Raw scores for neuropsychological tests were transformed into age-equivalent T-scores (M = 50; SD = 10)

Self-reported cognitive functioning is listed in Tables 4 and 5. In terms of executive difficulties as measured by BRIEF-A, the group with hydrocephalus reported greater difficulties on both Index scores, approximately one standard deviation on each Index. In terms of subscale level, no scores above clinical cut-off were seen in the group without hydrocephalus, whereas the group of five persons with hydrocephalus reported executive difficulties within the Emotional Control and Working Memory. Furthermore, on the three cognitive items on RPQ, three reported moderate problems with memory, four reported moderate problems with mental speed and one reported in the total sample. None reported severe problems. The group wise distribution is shown in Tables 4 and 5.

**Table 4:** Self-reported executive functioning.

	Total sample (N = 18)	Without hydrocephalus (N = 13)	With hydrocephalus (N = 5^a^)
	Mean (SD)	Mean (SD)	Mean (SD)
BRIEF-A			
• Global Executive Composite (GEC)	50.29 (9.69)	47.69 (8.50)	58.75 (9.36)
• Behavioral Regulation Index (BRI)	49.24 (9.34)	46.62 (8.07)	57.75 (8.85)
• Metacognitive Index (MI)	51.12 (9.29)	48.92 (8.72)	58.25 (8.22)

BRIEF-A: Behavior Rating Inventory of Executive Function for Adults

^a^BRIEF-A data from one participant in the group with hydrocephalus was excluded due to elevated score on the Inconsistency scale.

**Table 5:** Self-reported cognitive functioning.

	Total sample (N = 17)	Without hydrocephalus (N = 12)	With hydrocephalus (N = 5)
	N^a^	N^a^	N^a^
RPQ			
• Memory	3^a^	1^a^	2^a^
• Concentration	1^a^	0^a^	1^a^
• Mental speed	4^a^	2^a^	2^a^

RPQ: Rivermead Post-Concussion Symptom Questionnaire

^a^Number of participants reporting moderate (3) or severe (4) problems on RPQ

### Emotional distress

An overview of results on depression and anxiety for adults with and without hydrocephalus is provided in Table 6. The median score on PHQ-9 in the total sample was 6. Furthermore, six participants reported depressive symptoms corresponding to clinical levels of depression, and four reported to have been troubled with suicidal thoughts within the last 14 days. Median score on GAD-7 in the total sample was 5.0, in which five reported clinical levels of anxiety. Moreover, among those eleven who scored above cut-off for either depression or anxiety, three participants reported elevated clinical levels of both. The group wise distribution is shown in Table 6.

**Table 6:** Descriptive statistics. Depression, anxiety, resilience, coping and social support.

	Total sample (N = 19)	Without hydrocephalus (N = 14)	With hydrocephalus (N = 5)
	Median [Q1, Q3]/N	Median [Q1, Q3]/N	Median [Q1, Q3]/N
Patient Health Questionnaire 9 (PHQ-9)	6 [2, 11]	6 [1.8, 10.3]	6 [5, 20.5]
• Above cut-off (≥10)	6^a^	4^a^	2^a^
Generalized Anxiety Disorder 7 (GAD-7)	5 [3, 10]	3 [1, 5.5]	10 [7.5, 12.5]
• Above cut-off (≥10)	5^a^	2^a^	^a^ ^a^

PHQ-9: Patient Health Questionnaire; GAD-7: Generalised Anxiety Disorder

^a^Participants reporting symptom levels above cut-off

### Relationship between psychosocial factors and emotional distress

Table 7 displays the relationships between psychosocial factors and emotional distress. The resilience subscales perception of self and perception of future were significantly associated with symptoms of depression and anxiety. The correlation coefficients were all negative and ranged from medium to large [45], suggesting that higher degree of positive perception of self and future are moderately and strongly associated with lower symptoms of depression and anxiety. The remaining resilience subscales, social support, and coping strategies, did not correlate significantly with emotional distress.

**Table 7:** Relationship between psychosocial factors and emotional distress.

	Depression (PHQ-9 mean total score)	Anxiety (GAD-7 mean total score)
Resilience (RSA total score)	–.33	–.56*
• Perception of self	–.49*	–.72**
• Perception of future	–.60**	–.74**
• Social competence	–.35	–.34
• Family cohesion	.16	–.00
• Social resources	–.10	–.27
• Structured style	.14	.10
Social support (FSSQ mean total score)	.36	.40
Brief-Cope		
• Seeking social support	.34	.44
• Problem solving	.44	.12
• Avoidance	.29	.25
• Positive thinking	.24	.28

*p < .05

**p < .01

RSA: Resilience Scale for Adults; FSSQ: Duke-University of North Carolina Functional Social Support Questionnaire; Brief-Cope: Brief COPE Inventory; PHQ-9: Patient Health Questionnaire; GAD-7: Generalised Anxiety Disorder.

## Discussion

The present study aimed to describe cognitive function and emotional distress in adults with SBM aged 55 years and older. The studied population represents an emerging sub-group of individuals living into older age with SBM, and the current study explores important aspects of this understudied population that mainly have been investigated in children and young adults. All participants were born prior–or during the very first years of the establishment of shunt surgery for hydrocephalus in Norway. Therefore, they represent a selected group in several ways. Firstly, the group consists of long-term survivors into middle-age and older age, and secondly it is a “non-typical” SBM group with low prevalence of hydrocephalus in opposite to those born after shunt surgery became standard treatment.

Our main finding is that the majority of this study population with SBM displayed cognitive functioning within the normal range measured by both performance-based and self-reported measures, where fourteen participants did not have common SBM-related brain abnormalities. There was an observed discrepancy between participants with and without hydrocephalus, indicating lower cognitive functioning among the participants with hydrocephalus. This is in line with previous studies on cognitive functioning in adults. For example, Iddon et al. [47] investigated neuropsychological test performance in young adults with congenital hydrocephalus and/or SB. Whereas below-average test performance was seen in participants with hydrocephalus, participants with SB only did not differ from an included healthy control group, with the exception of semantic fluency [47]. Similarly, Bendt et al. [7] reported lower test performance in participants with SB and hydrocephalus regarding psychomotor function, mental speed, and executive functioning in their sample of 153 persons with SB with and without hydrocephalus aged 18–73 years. These findings indicate that SBM is not in itself associated with cognitive deficits, highlighting the importance of considering associated hydrocephalus, Chiari malformation type 2 and corpus callosum dysgenesis. A report from the International Federation for Spina Bifida and Hydrocephalus [48] proposed that the physical decline experienced by middle-aged and older persons living with SBM [5, 6] may represent accelerated ageing. Hence, one could expect a comparable cognitive decline, which was not indicated in this study.

In our cohort, six and five of nineteen participants had clinical levels of depressive and anxiety symptoms, respectively. The results were in accordance with previously reported prevalence rates in adults with SB [14, 15, 19]. Notably, the mean age in our cohort was approximately 20 years older than reported in the other study samples [14, 15, 19]. Emotional distress in the current cohort was screened with HADS in 2017 by Lidal et al. [20], in which about 1/5 and 1/3 scored above threshold for clinically relevant symptoms of depression and anxiety, respectively. The longitudinal results indicated a small increase in depressive symptom levels and a slight decrease in anxiety symptoms. However, comparisons between 2017 and 2021 is questionable. Firstly, different self-report instruments that operate with different cut-off values were applied. Secondly, the cohort is reduced from 30 to 19. Overall, our results showed that the majority of participants with SBM had good mental health, and that this seemed relatively stable into older age despite gradual physical decline. Interestingly, Schanke et al. [49] found overall satisfactory psychological health in older adults with poliomyelitis despite reporting to be quite unsatisfied with their physical health. Thus, the findings in the current study are in line with similar findings in other patient groups living with long-standing neurologic health conditions. On the other hand, although the majority in our sample reported good mental health, the results highlight the need to draw attention to those reporting emotional distress as well. The fact that four out of six participants that reported elevated symptom levels of depression expressed suicidal ideation is notably high and highlights the need for address suicidal risk screening in clinical practice. The results indicate, however, an important discrepancy between participants with and without hydrocephalus, with higher levels of emotional distress among the participants with hydrocephalus.

Our results indicate that perceptions of oneself and the future might also be worth considering in light of emotional distress, as the current study showed that these resilience factors were associated with lower levels of depression and anxiety symptoms. As mentioned, adults with SBM face challenges in terms of deteriorating health, reduced mobility, and secondary health complication, as well as unmet health care needs [5, 14]. These have been highlighted as potential mechanisms contributing to elevated psychological distress in SBM [15]. Consistent with our findings, Friborg et al. [50] demonstrated a strong association between perception of self and future and emotional stability. Similarly, in a qualitative study by King et al. [51], self-perception was mentioned as an important protective factor against adverse life events among participants with SB, cerebral palsy and attention deficit hyperactivity disorder. Even though SBM is a congenital condition, our findings are in accordance with other studies investigating resilience in adults with long-standing acquired disabilities, such as polio [49], multiple trauma [52], and spinal cord injury [52]. Even though social support and emotional distress yielded non-significant correlations in the current study, the coefficient was moderate. The lack of significance most likely represents lack of statistical power due to low sample size, as social support is known to buffer against emotional distress in both children and adults with SB [23, 24, 51] and individuals ageing with other somatic disabilities [53]. Also, Hayter et al. [19] found that social isolation predicted depression and anxiety in adults with SB.

This study has several limitations. It was limited by a small sample and cross-sectional design, which preclude conclusions regarding causality and limits statistical power. Due to this, the study is primarily descriptive and the correlation analysis were exploratory. Although results for the five persons with hydrocephalus were reported separately, the small sample precluded analyzing between-group differences statistically. Also, the lack of association between coping and social support and psychological distress might be a false negative, as some non-significant correlations were rather high. In addition, the study sample is a cohort that represent a subgroup of SB. Taking the reduction in number of participants from the original 2017-cohort along with the excluded participants due to health issues into account, cautions should be made regarding the generalizability as our study sample may be skewed towards healthier middle-aged adults with SBM living in Norway. Notwithstanding these limitations, this study represents one of very few studies exploring functional status in middle-aged and older persons with SB. The data are in need of replication in larger samples, but provide valuable information about this subgroup.

## Conclusion

This cross-sectional study provides insight in to an understudied SBM population consisting of a limited number of middle aged and older SBM survivors whom have lived a lifetime with their condition. Our results elucidate the heterogeneity in SBM by showing cognitive functioning within the normal range in this selected group of middle-aged and older adults. In addition, dividing the group with regard to hydrocephalus illustrated the importance of considering associated brain abnormalities when investigating cognitive functioning in SBM. The study furthermore supports previous research of emotional distress in adults with SBM, and indicates stable levels of emotional distress in the ageing SBM population despite having lived with long-standing health issues. However, the high proportion of participants reporting suicidal ideation reflect the need for addressing psychological health in clinical practice. In terms of protective psychosocial factors that might buffer against emotional distress, positive perceptions of self and future seemed to protect against depression and anxiety. Thus, clinical practice may benefit from strengthening the individual’s self-perception and have regular follow-ups. Future studies should aim to identify possible risk factors as well as protective factors for emotional distress in SBM.

## Supporting information

S1 Checklist STROBE statement–checklist of items that should be included in reports of observational studies. (DOCX)
