# Supplementary material for: Cognition and emotional distress in middle-aged and older adults with spina bifida myelomeningocele
Source: PLoS One. 2024 Feb 29;19(2):e0298891. doi: 10.1371/journal.pone.0298891 (PMC10903919; doi:10.1371/journal.pone.0298891)
Supplement: S1 Checklist — (DOCX) [file pone.0298891.s001.docx]

STROBE Statement—checklist of items that should be included in reports of observational studies

|  | Item No. | Recommendation | Page  No. | Relevant text from manuscript |
| --- | --- | --- | --- | --- |
| **Title and abstract** | 1 | (*a*) Indicate the study’s design with a commonly used term in the title or the abstract | 2 | Lines 30-32: “Cross-sectional study of eleven females and eight males with SBM, five with and twelve without hydrocephalus.” |
|  |  | (*b*) Provide in the abstract an informative and balanced summary of what was done and what was found | 2 | Lines 30-39: see sections “materials and method” and “results”. |
| Introduction | | | |  |
| Background/rationale | 2 | Explain the scientific background and rationale for the investigation being reported | 2-5 | Lines 49-117: The background is provided in the introduction section |
| Objectives | 3 | State specific objectives, including any prespecified hypotheses | 5 | Lines 119-122: “The primary aim of the current study was therefore to describe cognitive function and emotional distress in middle-aged and older adults with SBM. A secondary aim was to explore the associations between psychosocial factors, i.e. resilience, access to social support and coping in relation to emotional distress” |
| Methods | | | |  |
| Study design | 4 | Present key elements of study design early in the paper | 6 | Lines 128-129: “The target population in the current cross-sectional study was the same 30 persons (18 females and 12 males) from all parts of Norway with SB who participated in the Lidal et al. study in 2017.” |
| Setting | 5 | Describe the setting, locations, and relevant dates, including periods of recruitment, exposure, follow-up, and data collection | 6, 11 | Lines 136-144: See section “Participants and procedure”.  Line 248: “Statistical analyses were carried out in November 2022 using SPSS 28.0” |
| Participants | 6 | (*a*) *Cohort study*—Give the eligibility criteria, and the sources and methods of selection of participants. Describe methods of follow-up  *Case-control study*—Give the eligibility criteria, and the sources and methods of case ascertainment and control selection. Give the rationale for the choice of cases and controls  *Cross-sectional study*—Give the eligibility criteria, and the sources and methods of selection of participants | 6 | Lines 128-135: “The target population in the current cross-sectional study was the same 30 persons (18 females and 12 males) from all parts of Norway with SB who participated in the Lidal et al. study in 2017 [5]. This previous study recruited persons with SB born prior to 1967 who were registered at TRS National Resource centre for Rare Disorders at Sunnaas Rehabilitation Hospital, Norway. In addition, recruitment was conducted through announcement on the website of the Norwegian Association for Spina Bifida and Hydrocephalus (n=62). The exclusion criteria were psychiatric conditions that interfered with the ability to provide informed consent or co-morbid severe somatic illness that would interfere with participation.” |
|  |  | (*b*) *Cohort study*—For matched studies, give matching criteria and number of exposed and unexposed  *Case-control study*—For matched studies, give matching criteria and the number of controls per case | N/A |  |
| Variables | 7 | Clearly define all outcomes, exposures, predictors, potential confounders, and effect modifiers. Give diagnostic criteria, if applicable | 9, 11 | Description all outcomes provided in Method section under “Measurements”. Diagnostic criteria are shown in lines 199-200: “In line with recommendations, a score of 10 or above indicates clinically relevant depression.”;  lines 207-208: “A score of 10 is proposed as threshold for clinically relevant anxiety.”; and  lines 240-243: “Neuropsychological test performance was classified as “normal” if T-scores fell within 1½ SD from the mean. With regard to the BRIEF-A, age-corrected T-scores (M=50; SD=10) are provided for each index and subscale, in which clinically elevated scores were defined as a T-score of 65 or greater, in line with the manual.” |
| Data sources/ measurement | 8* | For each variable of interest, give sources of data and details of methods of assessment (measurement). Describe comparability of assessment methods if there is more than one group | 6-10 | Each variable of interest is described under the section “Measurements” |
| Bias | 9 | Describe any efforts to address potential sources of bias | 6 | Effort to prevent selection bias is stated in lines 140-142: “Four participants were precluded to travel a long distance to the hospital due to health issues and were offered a home visit.” |
| Study size | 10 | Explain how the study size was arrived at | 6 | Lines 128-133: “The target population in the current cross-sectional study was the same 30 persons (18 females and 12 males) from all parts of Norway with SB who participated in the Lidal et al. study in 2017 [5]. This previous study recruited persons with SB born prior to 1967 who were registered at TRS National Resource centre for Rare Disorders at Sunnaas Rehabilitation Hospital, Norway. In addition, recruitment was conducted through announcement on the website of the Norwegian Association for Spina Bifida and Hydrocephalus (n=62).”  Lines 135-137: “The cohort represented about 30% of persons with SBM aged 50+ alive in Norway in 2017 [5]. At the time of inclusion in the current study, four persons from the original cohort were deceased, leaving a total of 26 whom received a letter of invitation to participate primo 2021.” |

| Quantitative variables | 11 | Explain how quantitative variables were handled in the analyses. If applicable, describe which groupings were chosen and why | 10 | Lines 231-234: “Descriptive statistics were applied to depict frequencies. Due to a small sample size, descriptive statistics are reported in both median (Mdn) with 1st and 3rd quartile (Q1, Q3), mean (M) with standard deviation (SD), and range. Spearman Rho correlation coefficients were used to explore the relationship between psychosocial factors and emotional distress.” |
| --- | --- | --- | --- | --- |
| Statistical methods | 12 | (*a*) Describe all statistical methods, including those used to control for confounding | 10-11 | Lines 231-246: see section “Statistical analyses”. |
|  |  | (*b*) Describe any methods used to examine subgroups and interactions | 10 | Line 236-237: Participants with and without hydrocephalus are reported separately.” |
|  |  | (*c*) Explain how missing data were addressed | 11  13 | Lines 247: “Missing data on questionnaires were handled by imputing the missed item with the mean.”  For the neuropsychological tests, see left colon in Table 3 page 13. |
|  |  | (*d*) *Cohort study*—If applicable, explain how loss to follow-up was addressed  *Case-control study*—If applicable, explain how matching of cases and controls was addressed  *Cross-sectional study*—If applicable, describe analytical methods taking account of sampling strategy | N/A |  |
|  |  | (*e*) Describe any sensitivity analyses | N/A |  |
| Results | | | | |
| Participants | 13* | (a) Report numbers of individuals at each stage of study—eg numbers potentially eligible, examined for eligibility, confirmed eligible, included in the study, completing follow-up, and analysed | 11 | Lines 255-258: “Of the 26 eligible participants, four did not return the informed consent letter, and three were excluded due to severe comorbid health conditions or practical implications. (..) . The current study sample thus consists of 19 adults with SBM aged 55-68 years from the original cohort of 30.” |
|  |  | (b) Give reasons for non-participation at each stage | 11 | Lines 255-260: “Of the 26 eligible participants, four did not return the informed consent letter, and three were excluded due to severe comorbid health conditions or practical implications. (..) . The current study sample thus consists of 19 adults with SBM aged 55-68 years from the original cohort of 30. All responded to questionnaires, except from one, missing the RSA. Seventeen participants completed neuropsychological testing, although not all 17 were able to complete the full test battery.” |
|  |  | (c) Consider use of a flow diagram | 11 | See Fig 1. |
| Descriptive data | 14* | (a) Give characteristics of study participants (eg demographic, clinical, social) and information on exposures and potential confounders | 12 | Table 2 present demographic (sex, age, education, occupational status) and clinical variables (lesion level, hydrocephalus, ambulation status). |
|  |  | (b) Indicate number of participants with missing data for each variable of interest | 11 | Lines 258-260: “All responded to questionnaires, except from one missing the RSA and two missing the RPQ. Seventeen participants completed neuropsychological testing, although not all 17 were able to complete the full test battery.” |
|  |  | (c) *Cohort study*—Summarise follow-up time (eg, average and total amount) | N/A |  |
| Outcome data | 15* | *Cohort study*—Report numbers of outcome events or summary measures over time | N/A |  |
|  |  | *Case-control study—*Report numbers in each exposure category, or summary measures of exposure | N/A |  |
|  |  | *Cross-sectional study—*Report numbers of outcome events or summary measures | 12-15 | See Table 3, 4, 5, and 6 |
| Main results | 16 | (*a*) Give unadjusted estimates and, if applicable, confounder-adjusted estimates and their precision (eg, 95% confidence interval). Make clear which confounders were adjusted for and why they were included | N/A |  |
|  |  | (*b*) Report category boundaries when continuous variables were categorized | 9, 11 | See dichotomised variables in method section: lines 199-200 for depression; lines 207-208 for anxiety; and lines 240-243 for cognitive functioning. |
|  |  | (*c*) If relevant, consider translating estimates of relative risk into absolute risk for a meaningful time period | N/A |  |

Continued on next page

| Other analyses | 17 | Report other analyses done—eg analyses of subgroups and interactions, and sensitivity analyses | N/A |  |
| --- | --- | --- | --- | --- |
| Discussion | | | | |
| Key results | 18 | Summarise key results with reference to study objectives | 17-19 | Lines 348-403: See Discussion |
| Limitations | 19 | Discuss limitations of the study, taking into account sources of potential bias or imprecision. Discuss both direction and magnitude of any potential bias | 19 | Lines 404-416: A description of limitations is provided in the last paragraph. |
| Interpretation | 20 | Give a cautious overall interpretation of results considering objectives, limitations, multiplicity of analyses, results from similar studies, and other relevant evidence | 17-19 | Lines 348-416: See discussion |
| Generalisability | 21 | Discuss the generalisability (external validity) of the study results | 19 | Lines 410-413: “Taking the reduction in number of participants from the original 2017-cohort along with the excluded participants due to health issues into account, cautions should be made regarding the generalizability as our study sample may be skewed towards healthier middle-aged adults with SBM living in Norway.” |
| Other information | |  | | |
| Funding | 22 | Give the source of funding and the role of the funders for the present study and, if applicable, for the original study on which the present article is based | N/A |  |

*Give information separately for cases and controls in case-control studies and, if applicable, for exposed and unexposed groups in cohort and cross-sectional studies.

**Note:** An Explanation and Elaboration article discusses each checklist item and gives methodological background and published examples of transparent reporting. The STROBE checklist is best used in conjunction with this article (freely available on the Web sites of PLoS Medicine at http://www.plosmedicine.org/, Annals of Internal Medicine at http://www.annals.org/, and Epidemiology at http://www.epidem.com/). Information on the STROBE Initiative is available at www.strobe-statement.org.
